# Nursing care for cytoreduction and hyperthermic intraoperative chemotherapy in an Intensive Care Unit: a scoping review

**DOI:** 10.1590/1980-220X-REEUSP-2024-0176en

**Published:** 2024-11-25

**Authors:** Aline Branco, Fernanda Cirne Lima Weston, Giovanna da Rosa Soares, Graciele Fernanda da Costa Linch, Rita Catalina Aquino Caregnato

**Affiliations:** 1 Universidade Federal de Ciências da Saúde de Porto Alegre, Porto Alegre, RS, Brazil.

**Keywords:** Hyperthermic Intraperitoneal Chemotherapy, Intensive Care Units; Postoperative Care, Enhanced Recovery After Surgery, Nursing Care, Quimioterapia Intraperitoneal Hipertérmica, Unidades de Cuidados Intensivos, Cuidados Posoperatorios; Recuperación Mejorada Después de la Cirugía, Atención de Enfermería

## Abstract

**Objective:**

To map postoperative nursing care for critically ill adult and older patients admitted to the Intensive Care Unit after cytoreduction surgery with hyperthermic intraoperative intraperitoneal chemotherapy.

**Method:**

TScoping review according to the JBI methodology, with articles extracted from databases and gray literature, with no language or publica-tion date delimitation. The studies selection and results extraction process was carried out by two independent reviewers, using the soft-ware *EndNote*® and *Rayyan*®. PRISMA *Extension for Scoping Review* was used for the writing, with registration on the *Open Science Framework*.

**Results:**

Forty-two studies were selected. The analysis revealed 72 types of care grouped into 14 care areas. The use of an epidural catheter for anal-gesia, optimization of individualized hemodynamic status, and strict control of fluid balance were the most cited care measures.

**Conclusion:**

The mapping identified post-operative nursing care similar to those for major surgeries for patients recovering in the Intensive Care Unit, with an indication of the use of personal protective equipment by professionals when handling tubes in the first 48 hours of admission.

## RESUMEN

**Objetivo:**

Mapear los cuidados de enfermería postoperatorios para pacientes adultos y ancianos críticamente enfermos ingresados en la Unidad de Cuidados Intensivos después de una cirugía citorreductora con quimioterapia intraperitoneal hipertérmica.

**Método:**

Revisión del alcance según metodología JBI, con artículos extraídos de bases de datos y literatura gris, sin delimitación de idioma ni fecha de publicación. El proceso de selección de estudios y extracción de resultados fue realizado por dos revisores independientes, a través de los programas *EndNote* ^®^ y *Rayyan* ^®^. Se utilizó PRISMA. *Extension for Scoping Reviews* para la redacción, registrándose en el *Open Science Framework*.

**Resultados:**

Se seleccionaron 42 estudios. El análisis mostró 72 tipos de atención agrupados en 14 áreas de atención. El uso de un catéter epidural para analgesia, la optimización del estado hemodinámico individualizado y el control estricto del balance hídrico fueron las medidas citadas con mayor frecuencia.

**Conclusión:**

El mapeo identificó cuidados de enfermería postoperatorios similares a los de cirugía mayor para pacientes recuperados en la Unidad de Cuidados Intensivos, con indicaciones para el uso de equipos de protección personal para los profesionales en la manipulación de drenajes en las primeras 48 horas de ingreso.

## INTRODUCTION

In recent decades, there has been an increase in the incidence of several neoplasms, requiring a constant search for new types of oncological treatments. Among the surgical approaches, cytoreduction associated with hyperthermic intraperitoneal chemotherapy stands out (HIPEC), developed in the 1980s and indicated for the removal of malignant abdominal tumors in reproductive, gastrointestinal, and peritoneal organs^(1, 2, 3)^.

Cytoreduction is characterized by the manual removal of visible tumor and perilesional sites by the surgeon; meanwhile, HIPEC, commonly performed at the same intraoperative moment, consists of the application of chemotherapy at a temperature of 40 to 43ºC in the abdominal cavity, being kept in place for 60 to 120 minutes, to allow its direct action on the organs affected by the neoplasm^(4,5)^. Chemotherapy high temperature is essential, as it stimulates the immune response, the direct action of the drug on neoplastic areas, and the apoptosis of tumor cells in the body^(6,7)^.

The combination of both procedures results in a highly complex surgery; therefore, patients are referred to the Intensive Care Unit (ICU) for post-operative recovery. The effectiveness of the treatment does not rely only on the application of the correct surgical technique, but also on adequate post-operative management by the multidisciplinary team. Currently, international recommendations from the *Enhanced Recovery After Surgery* (ERAS), specific to HIPEC-associated cytoreduction, have a section dedicated exclusively to postoperative care^(8,9)^. ERAS aims to bring crucial points of care that must be implemented for patients from the first day after surgery, to make care more efficient and advance patients’ full recovery^(9)^.

Among the main practices recommended by ERAS are the removal of urinary catheter until the 3rd postoperative day, analgesia via epidural catheter, early mobilization and introduction of clear liquids on the first postoperative day^(8)^, all carried out by the multidisciplinary team within the ICU care routine. ICU nursing, part of the multidisciplinary team, provides continuous bedside care and assumes responsibility for a significant part of post-operative care. The scope of this care includes adequate pain management, preferably through epidural analgesia, based on constant pain assessments; administration of antiemetics; early mobilization; constant glycemic and thermal monitoring; implementation of adequate fluid balance; care with tubes^(8,9,10)^.

Although the importance of postoperative nursing care in the ICU for the therapeutic success of HIPEC-associated cytoreduction is recognized, studies addressing it are still scarce. The role of intraoperative nursing in assisting patients during surgery, through the manipulation of chemotherapy infusion devices, assistance with anesthetic, care and protection of professionals during the HIPEC phase, is addressed in the literature^(11,12)^. A Brazilian study on postoperative care for patients undergoing HIPEC was highlighted, but in the format of a narrative literature review^(13)^.

Most research emphasizes medical procedures, which represent a barrier to establishing care protocols to benefit these patients^(8,9,14)^. Specific protocols and guidelines for the nursing team were also not identified, which may hinder the standardization of care and implementation of evidence-based practice in the ICU^(13)^. Therefore, a gap in nursing knowledge is observed regarding postoperative care in patients undergoing HIPEC-associated cytoreduction. Therefore, it was decided that a mapping of the literature would be carried out to identify which postoperative care is recommended for these patients according to the scientific literature. This article aims to map postoperative nursing care for critically ill adult and older patients admitted to the ICU after HIPEC-associated cytoreduction surgery.

## METHOD

### Design of Study

A scoping review was carried out because it is the recommended method for mapping evidence, aimed at identifying the state of the art on concepts and theories, available evidence, and at finding knowledge gaps^(15)^. To develop this review, the JBI guidelines were followed, based on these steps: identification of the research question; verification of relevant studies in the sources of evidence; selection of studies to be included in the review according to eligibility criteria; mapping and extraction of data from the studies included in the review; and organization and presentation of the results^(16)^. To ensure the quality and transparency of the writing, PRISMA *Extension for Scoping Review* (PRISMA-ScR) was used, with analysis of whether all review development items were covered^(17)^. A preliminary search in PROSPERO, MEDLINE, *Cochrane Database of Systematic Reviews*, and JBI *Evidence Synthesis* was carried out, and no ongoing protocols, systematic reviews or scoping reviews on the topic were found. A protocol for scope review was developed*,* registered in *Open Science Framework* (OSF) under DOI 10.17605/OSF.IO/UZH5K.

### Data Collection

In the first stage, the research question was structured using the mnemonic PCC^(15,16)^ where: (P)opulation refers to adult and elderly cancer patients undergoing the surgical procedure of cytoreduction with HIPEC who recover in the ICU; (C)ontext, studies whose context is exclusively the Intensive Care Unit environment and; (C)oncept, postoperative nursing care necessary for the recovery of these patients, generating the question “what postoperative nursing care is indicated for critical adult and elderly patients admitted to the ICU after cytoreduction surgery with HIPEC?”.

The research strategy was carried out in three stages. Initially, studies on postoperative care for cytoreduction and HIPEC were searched in PubMed (MEDLINE) to verify which terms were most commonly used. These terms were searched for in the format of descriptors indexed in Portuguese in the Health Sciences Descriptors (DeCS) and in English in the *Medical Subject Headings (MeSH).* After selecting the indexed descriptors, two initial search strategies were developed for PubMed (MEDLINE) and CINAHL, to verify whether they included studies that answered the research objective and question, with the search carried out on August 5, 2023, returning 372 articles, as shown in Chart 1. Access to the strategies developed for the other databases can be consulted in full in the scoping review protocol registered with the OSF.

**Chart 1:** – Search strategies for the PubMed (MEDLINE) and CINAHL databases - Porto Alegre, RS, Brazil, 2024.

Database	Search strategy	Results
**PubMed (MEDLINE)**	(Cytoreduction Surgical Procedures[mh] OR Cytoreduct*[tiab] OR Debulk*[tiab]) AND (Hyperthermic Intraperitoneal Chemotherapy[mh] OR Hyperthermia, Induced[mh:noexp] OR Hyperthermic Intraperitoneal Chemotherap*[tiab] OR HIPEC[tiab] OR Hot Chemotherap*[tiab] OR Intraperitoneal Hyperthermic Chemotherap*[tiab] OR Induced Hypertherm*[tiab] OR Therapeutic Hypertherm*[tiab] OR Thermotherap*[tiab] OR Fever Therap*[tiab] OR Local Hypertherm*[tiab]) AND (Intensive Care Units[mh:noexp] OR Respiratory Care Units[mh] OR Critical Care[mh] OR Critical Care Nursing[mh] OR Intensive care*[tiab] OR Critical care*[tiab] OR ICU[tiab] OR Care Unit*[tiab] OR Nursing Care[mh:noexp] OR Medical-Surgical Nursing[mh] OR Oncology Nursing[mh] OR Nurses[mh] OR Nursing[tiab] OR Nurse*[tiab] OR Postoperative Period[mh] OR Postoperative Care[mh] OR Recovery Room[mh] OR Enhanced Recovery After Surgery[mh] OR Postoperat*[ti] OR Post-operat*[ti] OR Post-surg*[ti] OR Recover*[ti])	315
**CINAHL**	(MH “Cytoreduction Surgical Procedures” OR TI ( Cytoreduct* OR Debulk* ) OR AB ( Cytoreduct* OR Debulk* ) OR SU ( Cytoreduct* OR Debulk* ) AND (MH ( “Hyperthermic Intraperitoneal Chemotherapy” OR “Hyperthermia, Induced” ) OR TI ( “Hyperthermic Intraperitoneal Chemotherap*” OR “HIPEC” OR “Hot Chemotherap*” OR “Intraperitoneal Hyperthermic Chemotherap*” OR “Induced Hypertherm*” OR “Therapeutic Hypertherm*” OR “Thermotherap*” OR “Fever Therap*” OR “Local Hypertherm*” ) OR AB ( “Hyperthermic Intraperitoneal Chemotherap*” OR “HIPEC” OR “Hot Chemotherap*” OR “Intraperitoneal Hyperthermic Chemotherap*” OR “Induced Hypertherm*” OR “Therapeutic Hypertherm*” OR “Thermotherap*” OR “Fever Therap*” OR “Local Hypertherm*” ) OR SU ( “Hyperthermic Intraperitoneal Chemotherap*” OR “HIPEC” OR “Hot Chemotherap*” OR “Intraperitoneal Hyperthermic Chemotherap*” OR “Induced Hypertherm*” OR “Therapeutic Hypertherm*” OR “Thermotherap*” OR “Fever Therap*” OR “Local Hypertherm*” ) AND (MH ( “Intensive Care Units” OR “Respiratory Care Units” OR “Critical Care” OR “Critical Care Nursing” OR “Nursing Care” OR “Medical-Surgical Nursing” OR “Oncology Nursing” OR “Nurses” OR “Postoperative Period” OR “Postoperative Care” OR “Recovery Room” OR “Enhanced Recovery After Surgery” ) OR TI ( “Intensive care*” OR “Critical care*” OR “ICU” OR “Care Unit*” OR “Nursing” OR “Nurse*” OR “Postoperat*” OR “Post-operat*” OR Post-surg* OR Recover* ) OR AB ( “Intensive care*” OR “Critical care*” OR “ICU” OR “Care Unit*” OR “Nursing” OR “Nurse*” ) OR SU ( “Intensive care*” OR “Critical care*” OR “ICU” OR “Care Unit*” OR “Nursing” OR “Nurse*” )	57

Note: Pubmed - National Library of Medicine; CINAHL - Cumulative Index to Nursing and Allied Health Literature.

The initial strategies were later adapted for the databases Virtual Health Library (VHL), Scopus (Elsevier), Embase (Elsevier), CINAHL (Ebsco), CENTRAL (John Wiley) and Web of Science (Clarivate Analytics). Access to Scopus, Embase, CENTRAL and *Web of Science* databases took place through the Periodicals Portal, of the Coordination for the Improvement of Higher Education Personnel (CAPES) in Brazil, while CINAHL through Ebsco, with search for articles in all databases from August 26 to 30, 2023. Furthermore, the reference lists of the articles included in the scoping review were consulted for relevant studies, searches for theses and dissertations were carried out in the ProQuest database, and for gray literature in the *System for Information on Gray Literature in Europe, British Library EThOS ,* and *Center for Reviews and Dissemination* (*York University*). The search for descriptors and final development of the strategies were carried out by a librarian specialized in research in the health area. The entire process of selection of the studies included in the review until data extraction was done by two researchers in a concealed way, and in the event of divergence in the studies included or results extracted, a third reviewer was called upon.

### Eligibility Criteria

Primary studies using qualitative and quantitative methodology, review studies, theses, dissertations, and book chapters available in full electronic format were chosen as sources of information. Letters, opinion articles, editorials and scientific abstracts, duplicate documents, as well as studies that covered the intra- and transoperative periods and that had children and adolescents as their target audience were excluded. There were no restrictions on year of publication or language. It was decided not to restrict the period, as when developing the first search strategies, few studies addressing the topic were observed. The initial selection of studies occurred by excluding duplicates, reading the title and abstract. Once the theme and research question had been considered, the text was read in full. The articles were initially exported to the *EndNote* ^®^ where duplicates were removed, and then selected using the *Rayyan* ^®^. For those read in full, two researchers independently recommended inclusion and exclusion, and when there was disagreement a third reviewer was called upon.

### Data Extraction And Analysis

The authors developed their own instrument based on that recommended by the JBI for extracting data from studies considered for the scoping review*,* consisting of title, author, year, journal, country, research objective, methodological design, population, and care of ICU patients undergoing HIPEC-associated cytoreduction. Among the care measures, information was extracted regarding hemodynamic and ventilatory monitoring; assessment and assistance with patient pain; neurological pattern; care with surgical wounds, drains, probes, ostomy, tubes and catheters; prevention and rational management of healthcare-associated infections (HAIs); glycemic, thermal and nutritional control; mobilization; care with stress ulcer prophylaxis and deep vein thrombosis; mental health care and family attention; and occupational care with the team regarding exposure to chemotherapy. These care areas to be explored, regarding the respective mapped care, were determined due to the previous experience of the main author who provides care to patients undergoing cytoreduction and HIPEC in the ICU.

Data were documented in an Excel spreadsheet^®^ for better extraction of results and data evaluation. A descriptive analysis was carried out in terms of the percentage of frequency of citation of a given care, as well as the profile of the publications. Subsequently, the mapped results were grouped according to the care object.

### Ethical Aspects

As this is a scoping review study using publicly available secondary data, approval by the Research Ethics Committee is not required. The criteria of authorship of the authors and veracity of the information were followed.

## RESULTS

**Figure 1:**
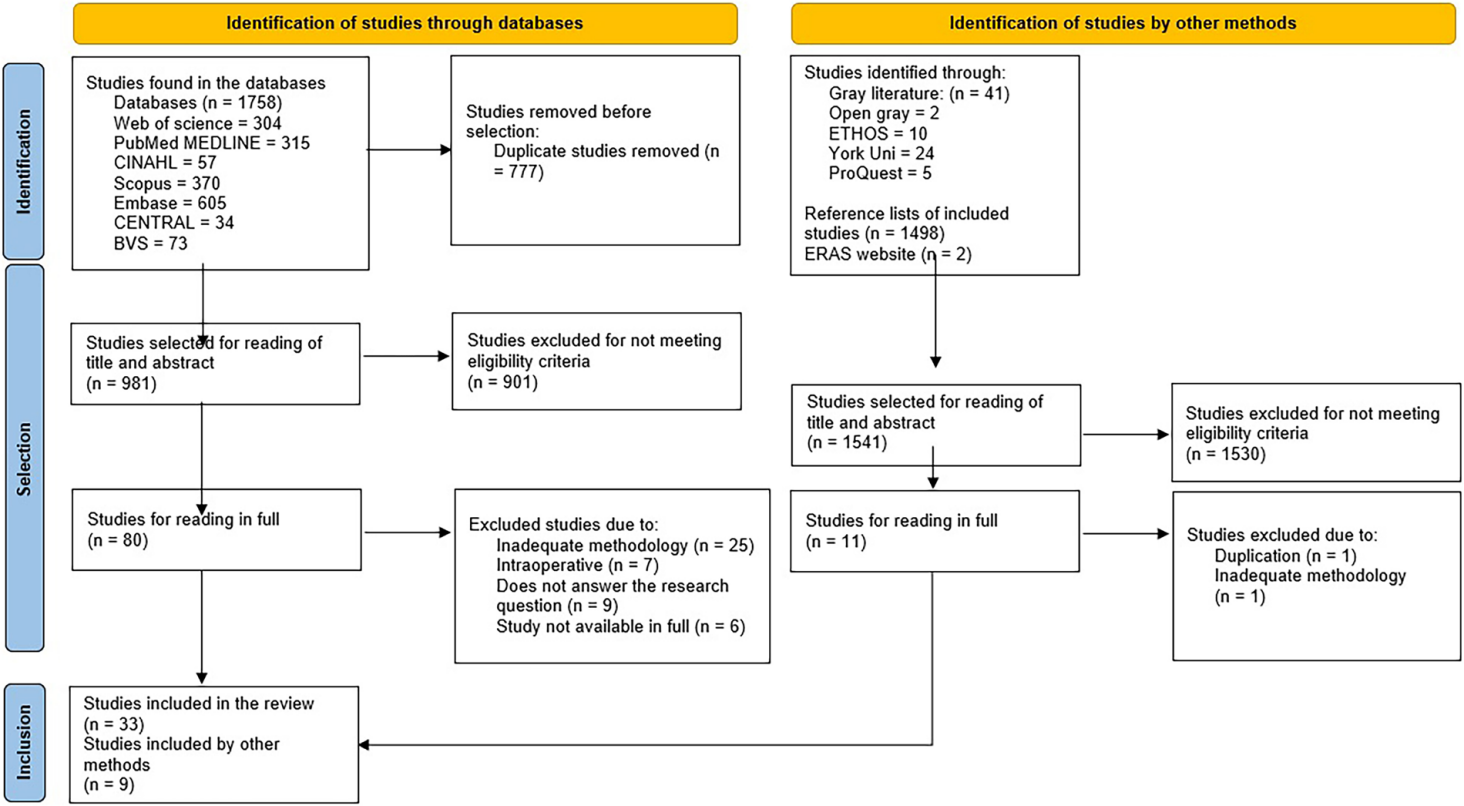
PRISMA flowchart of the article selection process. Porto Alegre, RS, Brazil, 2024.

Note: Pubmed – National Library of Medicine; CINAHL – Cumulative Index to Nursing and Allied Health Literature; VHL – Virtual Health Library.

During the study selection process, 1,758 articles were initially returned as results. After excluding duplicate studies and applying the eligibility criteria, a total of 42 studies resulted, with 40 scientific articles and two book chapters included in the scoping review, as shown in Figure 1.

The publication period ran from 2008 to 2022, with the year 2019^(4,14,18,19,20,21^ having the higher prevalence of publications. The highest concentration of publications was observed in the journal *European Journal of Surgical Oncology* ^(7,8,23,24,25)^, with authorships concentrated most prevalently in the United States (n = 14), India (n = 5), Germany (n = 4), Italy (n = 3), China (n = 3), Sweden (n = 3), Brazil (n = 2), Spain (n = 2), and one article in the following countries: France, Türkiye, England, Denmark, Poland, and Ireland. Furthermore, the vast majority of results comprised narrative literature reviews (n = 16), followed by cross-sectional observational studies (n = 8) and retrospective cohort studies (n = 8), systematic reviews (n = 3), qualitative studies (n = 3), book chapters (n = 2), case studies (n = 1), and experience reports (n = 1).

The main reason for exclusion from the sample was inadequate methodology (53%), referring to articles that were presented in the format of scientific abstracts published in annals. Regarding the databases of the 42 results included in the review, PubMed (60%) concentrated the majority of studies, followed by SCOPUS and *Web of Science* (10%), reference lists (21%), Embase (7%), and CINAHL (2%). Aiming at mapping the postoperative nursing care to be performed on patients undergoing cytoreduction and HIPEC with recovery in the ICU, the authors decided to characterize the care in specific areas of patient care needs in the postoperative period. Through the application of a specific data extraction instrument, 177 care procedures addressed individually by the studies were initially observed.

As the care measures were repeated in the studies included in the scope review, they were grouped into their care areas, referencing their respective cited studies. Thus, from the assistance needs initially mapped, the final result was 72 care needs. Nursing care mapped in the postoperative period in the ICU for patients undergoing cytoreduction and HIPEC can be seen in Chart 2.

**Chart 2:** Care areas and their respective nursing care for patients undergoing cytoreduction and HIPEC with postoperative recovery in the ICU – Porto Alegre, RS, Brazil, 2024.

Assistance areas	Mapped care	Presentation of studies
**Hemodynamic Monitoring**	Observe the need for fluid replacement, transfusion of blood products, electrolytes and initiation of inotropes or vasopressors according to the individualized hemodynamic assessment^(1,2,4,5,7,9,10,14,26,27,28,29,30,31,32,33,34,35,36)^.	Systematic review^(4,9)^, cross-sectional study^(10,28,31,35)^, narrative literature review^(2,5,7,14,26,27,29,32,34,36)^, experience report^(1)^, book chapter^(30,33)^.
	Strictly control fluid balance, monitoring fluid gains and losses through thoracic and abdominal drains, nasogastric/nasoenteric tubes, and urinary catheter/spontaneous diuresis^(1,2,4,5,7,13,27,29,30,32,33,34,36,37)^.	Systematic review^(4)^, narrative literature review^(2,5,7,13,27,29,32,34,36,37)^, experience report^(1)^, book chapter^(30,33)^.
	Evaluate laboratory tests: observing electrolyte values, coagulation profile, hematocrit and hemoglobin, acid-base balance, lactate, and other relevant tests^(2,4,5,13,14,29,30,36,37,38)^.	Systematic review^(4)^, cross-sectional study^(38)^, narrative literature review^(2,5,13,14,29,30,36,37)^.
	Monitor for signs of bleeding and hemorrhage^(3,5,7,29,30)^.	Narrative literature review^(5,7,29)^, case study^(3)^, book chapter^(30)^.
	Monitor vital signs and hemodynamic patterns, based on assessment of invasive mean arterial pressure, central venous pressure, cardiac output and index, and heart rate, in addition to peripheral perfusion^(1,13,14,29,33,37,38)^.	Cross-sectional study^(38)^, experience report^(1)^, narrative literature review^(13,14,29,37)^, book chapter^(33)^.
	Pay attention to kidney function, observing urinary volume and evaluating laboratory tests for urea and creatinine, observing signs of edema, measuring body weight and imaging tests^(1,5,7,9,13,26,28,30,32,36)^.	Systematic review^(9)^, cross-sectional study^(28)^, experience report^(1)^, narrative literature review^(5,7,13,26,32,36)^, book chapter^(30)^.
	Observe the need for monitoring intra-abdominal pressure, mainly for patients who had pressure above 12 cmH2O during surgery^(39)^.	Cross-sectional study^(39)^.
**Mechanical Ventilation and Oxygen Therapy**	Assess the need to keep the patient on invasive mechanical ventilation in the ICU, considering hemodynamic instability and complications related to the intraoperative and postoperative periods^(2,4,13,14,26,29,32,37,38)^.	Systematic review^(4)^, cross-sectional study^(38)^, narrative literature review^(2,13,14,26,29,32,37)^.
	Opt for early weaning and extubation as soon as possible in the operating room or ICU^(1,2,4,14,28,29,31,32,33)^.	Systematic review^(4)^, cross-sectional study^(28,31)^, experience report^(1)^, narrative literature review^(2,14,29,32)^, book chapter^(33)^.
	After extubation, opt for non-invasive ventilation such as continuous positive airway pressure (CPAP) or high-flow nasal cannula (HFNC), with the multidisciplinary team^(4,7,25,27,29,32,33)^.	Systematic review^(4)^, retrospective cohort study^(25)^, narrative literature review^(7,27,29,32)^, book chapter^(33)^.
	Perform bronchial hygiene by aspirating secretions from the airways and stimulating coughing, along with deep breathing exercises and spirometry^(5,9,13,33,40)^.	Systematic review^(9)^, cross-sectional study^(40)^, narrative literature review^(5,13)^, book chapter^(33)^.
	Observe signs of ventilatory complications in the postoperative period^(13,26,31,32,37,38)^.	Cross-sectional study^(31,38)^, narrative literature review^(13,26,32,37)^.
	Use a nasogastric tube for decompression and avoid the risk of aspiration^(37)^.	Narrative literature review^(37)^.
	Monitor respiratory pattern, with lung auscultation and documentation of sounds every 4 hours^(1,2,13,28,31,33,36,37,38)^.	Cross-sectional study^(28,31,38)^, experience report^(1)^, narrative literature review^(2,13,36,37)^, book chapter^(33)^.
**Neurological Pattern**	Monitor the patient’s awakening process^(41)^.	Cross-sectional observational study^(41)^.
**Patient Pain Assessment and Care**	Use multimodal analgesia: combination of local anesthetics or opioids in the epidural catheter with nonsteroidal anti-inflammatory drugs or other analgesics^(2,8,9,10,14,18,19,20,25,40,42)^.	Systematic review^(8,9)^, cohort study^(19,25,40,42)^, cross-sectional study^(10,20)^, narrative literature review^(2,14,18)^.
	Avoid the use of intravenous opioids by using other forms of analgesia such as an epidural catheter^(8,9,10,27,28)^.	Systematic review^(8,9)^, cross-sectional study^(10,28)^, narrative literature review^(27)^.
	Establish specialized services for acute pain, in the “Pain Service” modality. In cases of chronic pain, contact the Palliative Care team^(26,32,42)^.	Cohort study^(42)^, narrative literature review^(26,32)^.
	Perform analgesia through an epidural catheter as a safe method for pain control^(4,7,8,9,14,18,19,20,24,25,26,27,29,31,32,33,34,36,38,40,41,42,43)^.	Systematic review^(4,8,9)^, cohort study^(19,25,40,42)^, cross-sectional study^(20,31,38,41)^, narrative literature review^(7,14,18,24,26,27,29,32,34,36,43)^, book chapter^(33)^.
	Keep the epidural catheter in use until the transition of analgesia to oral or IV and discontinuation of the catheter^(4,8,13,19,24,25,26,36,40,42)^.	Systematic review^(4,8)^, cohort study^(19,25,40,42)^, narrative literature review^(13,24,26,36)^.
	Maintain assessment and care for pain relief, documenting it every 4 hours using appropriate scales^(3,13,33)^.	Case study^(3)^, narrative literature review^(13)^, book chapter^(33)^.
	Manage pain through Patient Controlled Analgesia (PCA) or transversus abdominis plane block (TAP) by the anesthetist, associated with multimodal analgesia^(10,13,14,18,25,33,36,42,43)^.	Cohort study^(25,42)^, cross-sectional study^(10)^, narrative literature review^(13,14,18,36,43)^.
	Use PCA only with the patient awake and instructed on its use^(13,33)^.	Narrative literature review^(13)^, book chapter^(33)^.
	Keep the epidural catheter in use for pain relief for 72 to 96 hours, with some studies suggesting use up to the 5th and 8th day postoperatively^(4,8,13,14,24,25,26,36)^.	Systematic review^(4,8)^, cohort study^(25)^, narrative literature review^(13,14,24,26,36)^.
	Control analgesic infusion via epidural catheter according to hemodynamic and neurological patterns. Maintain an aseptic environment during handling and maintenance^(8,26,42)^.	Systematic review^(8)^, cohort study^(42)^, narrative literature review^(26)^.
**Surgical Wound**	Assess the surgical incision for signs of infection, keeping it protected with a sterile dressing, and observe for bleeding or the presence of fistulas^(5,33)^.	Narrative literature review^(5)^, book chapter^(33)^.
	To prevent dehiscence, instruct the patient to cough using a pillow while pressing firmly on the incision^(5,33)^.	Narrative literature review^(5)^, book chapter^(33)^.
	Assess negative pressure dressing for integrity and function^(36)^.	Narrative literature review^(36)^.
**Care of Drains, Ostomies, Probes, Tubes and Catheters**	Assess the volume and appearance of fluids drained through thoracic and abdominal drains and nasogastric or nasoenteric tubes^(2,5,13,26,33,37)^.	Narrative literature review^(2,5,13,26,37)^, book chapter^(33)^.
	Observe the appearance of secretion from ostomies, surgical wound and rectum. Inform the medical team if there are signs of content change^(13,37)^.	Narrative literature review^(13,37)^.
	Use dry sterile gauze to protect the skin around the drains^(33,36)^.	Narrative literature review^(36)^, book chapter^(33)^.
	Daily assess the need to maintain a nasogastric tube and drainage of residual content^(10,20)^.	Cross-sectional study^(10,20)^.
	Observe the volume of chest drains and the need for permanence. Reservoirs should never be more than half full, monitoring the volume every 1-2 hours^(13,33)^.	Narrative literature review^(13)^, book chapter^(33)^.
	Beware that the installation of a nasogastric tube is not recommended for all patients, if there is no risk of delayed gastric emptying due to omentum resection^(8,28)^.	Systematic review^(8)^, cross-sectional study^(28)^.
	Remove urinary catheter, nasogastric tube, thoracic and abdominal drains within 72 hours of hospitalization, or as soon as possible^(10,19,20,28)^.	Cohort study^(19)^, cross-sectional study^(10,20,28)^.
	Introduce the patient and family to ostomy care^(18)^.	Narrative literature review^(18)^.
**Rational Prevention and Management of Infections**	Properly clean hands with soap and water, especially before and after contact with the patient’s drains, secretions and surgical sites^(18,33)^.	Narrative literature review^(18)^, book chapter^(33)^.
	Involve family and patient in education about signs and symptoms of surgical site infection, and instruct on hand washing and use of personal protective equipment^(18)^.	Narrative literature review^(18)^.
	Maintain an aseptic environment for insertion of central catheters, using Doppler ultrasound to guide insertion, as well as adequate manipulation of the pathways and hand hygiene^(21,37)^.	Cohort study^(21)^, narrative literature review^(37)^.
	Use antibiotics rationally and scale them according to the results of appropriate cultures and the patient’s condition^(2,4,29)^.	Systematic review^(4)^, narrative literature review^(2,29)^.
	Pay attention to signs of infection due to changes in the composition of drainage in drains and the surgical wound^(5,23,37)^.	Cohort study^(23)^, narrative literature review^(5,37)^.
**Glycemic Control**	Maintain strict glycemic control. The target glucose value in critically ill patients should be between 140 – 180 mg/dL^(4,8,9,19,26,29)^.	Systematic review^(4,8,9)^, cohort study^(19)^, narrative literature review^(26,29)^.
	Pay attention to hyperglycemia, commonly observed, requiring the use of insulin, especially in patients undergoing interventions on the pancreas^(4,8,19,26)^.	Systematic review^(4,8)^, cohort study^(19)^, narrative literature review^(26)^.
	Assess the need for continuous insulin infusion to correct hyperglycemia^(9,29)^.	Systematic review^(9)^, narrative literature review^(29)^.
**Thermal Control**	Monitor temperature postoperatively, continuously and rigorously, using heated fluids and thermal blankets^(10,14,32)^.	Cross-sectional study^(10)^, narrative literature review^(14,32)^.
	Maintain normothermia (body temperature > 36°C) and evaluate serum lactate along with other inflammatory markers to assess tissue perfusion^(4,10,30,32)^.	Systematic review^(4)^, cross-sectional study^(10)^, narrative literature review^(32)^, book chapter^(30)^.
**Nutritional Control**	Give preference to oral nutrition. If there are abdominal complications in which the patient does not tolerate enteral nutrition, start parenterally if the nutritional delay exceeds more than three days^(4,7,9,14,18,36,37,38,44,45)^.	Systematic review^(4,9)^, cohort study^(44)^, cross-sectional study^(38)^, narrative literature review^(7,14,18,36,37,45)^.
	Start nutrition as soon as possible, providing clear fluids on the first day after surgery. Progress according to acceptance, tolerance, and clinical evaluation to a low-residue diet, and then to solid foods^(7,8,9,10,14,19,20,25,28,29,32,33,38,45)^.	Systematic review^(8,9)^, cohort study^(19,25)^, cross-sectional study^(10,20,28,38)^, narrative literature review^(7,14,29,32,45)^, book chapter^(33)^.
	Assess bowel function by listening for bowel sounds, flatulence, evacuation and gastric residue volume, documenting acceptance and weight gain^(8,9,13,24,28,33,36)^.	Systematic review^(8,9)^, cross-sectional study^(28)^, narrative literature review^(13,24,36)^, book chapter^(33)^.
	Observe the patient’s tolerance to the nutritional modality offered to identify those with insufficient nutrition and signs of complications^(18,24,33)^.	Narrative literature review^(18,24)^, book chapter^(33)^.
	Associate intravenous fluid supply for energy supply, if necessary ^(4,7,13,14,24,28,36,37,38)^.	Systematic review^(4)^, cross-sectional study^(28,38)^, narrative literature review^(7,13,14,24,36,37)^.
	Start total parenteral nutrition (TPN) as early as possible in case of signs of complications that prevent enteral route. The duration of TPN is based on nutritional status, complications, and caloric count^(4,7,30,36-38)^.	Systematic review^(4)^, cross-sectional study^(38)^, narrative literature review^(7,36,37)^, book chapter^(30)^.
	Avoid undue postoperative fasting for more than 24 hours, as this is a risk factor for prolonged ICU stay^(7,37,38,46)^.	Cohort study^(46)^, cross-sectional study^(38)^, narrative literature review^(7,37)^.
	Do not administer oral feeding (NPO), if at risk due to the surgical condition, until return of bowel sounds, flatulence and a decrease in gastric residues^(13,33)^.	Narrative literature review^(13)^, book chapter^(33)^.
	Use prokinetics, laxatives and chewing gum to prevent paralytic ileus^(8,19,28)^.	Systematic review^(8)^, cohort study^(19)^, cross-sectional study^(28)^.
**Mobilization**	Encourage early mobilization from the first postoperative day^(4,5,8,9,10,20,24,28,40)^.	Systematic review^(4,8,9)^, cohort study^(40)^, cross-sectional study^(10,20,28)^, narrative literature review^(5,24,40)^.
	Change the position in bed every two hours, taking care with the thoracic and abdominal drains^(13,33)^.	Narrative literature review^(13)^, book chapter^(33)^.
	Mobilize in bed on the first postoperative day and between the first and fourth postoperative day encourage getting out of bed to use an armchair and walk^(7-10,19,24,25,33,40)^.	Systematic review^(8,9)^, cohort study^(19,25,40)^, cross-sectional study^(10)^, narrative literature review^(7,24)^, book chapter^(33)^.
	Monitor drainage and drain preservation, surgical incision, and hemodynamic changes during mobilization^(38,41)^.	Cross-sectional study^(38,41)^.
**Stress Ulcer and DVT Prophylaxis**	Prevent stress ulcers by using H2 receptor antagonists and proton pump inhibitors, as prescribed by the doctor^(2,29,31)^.	Cross-sectional study^(31)^, narrative literature review^(2,29)^.
	Administer, as prescribed by the physician, pharmacological prophylaxis with low molecular weight heparin or unfractionated heparin, and use intermittent lower limb compressors and graduated compression stockings^(2,4,5,7,8,9,14,19,29,33)^.	Systematic review^(4,8,9)^, cohort study^(19)^, narrative literature review^(2,5,7,14,29)^, book chapter^(33)^.
	Start pharmacological prophylaxis 12 hours before surgery, extending until the fourth week of hospitalization or until complete mobilization^(4,8,9,19)^.	Systematic review^(4,8,9)^, cohort study^(19)^.
	Promote adequate hydration, early ambulation, leg exercises while in bed or chair^(33)^.	Book chapter^(33)^.
**Mental Health and Family Care**	Adjust nursing staffing to better care for this patient profile^(1)^.	Experience report^(1)^.
	Maintain direct, clear and effective communication with the patient and family, involving information about the surgical procedure, recovery process, expected symptoms and adverse effects, treatment plans and joint decision-making about therapy^(18,22,24,47,48)^.	Qualitative study^(22,47,48)^, narrative literature review^(18.24)^.
	Involve patient and family in educating them about the care that will be needed post-operatively and during the recovery process^(9,18,22,24)^.	Systematic review^(9)^, qualitative study^(22)^, narrative literature review^(18,24)^.
	Promote embracing, support for needs, humanized care, encourage the patient to face cancer and strengthen the bond of trust among the patient, family, and multidisciplinary team^(32,47,48)^.	Qualitative study^(47,48)^, narrative literature review^(32)^.
	Consider the need for psychological support and social assistance. Whenever possible, conduct a visit to the ICU before the procedure as part of guidance and to reduce post-operative anxiety^(24,25,32)^.	Cohort study^(25)^, narrative literature review^(24,32)^.
	Promote quality sleep, reduce noise in the ICU, and allow open visits by family members^(24,47)^.	Qualitative study^(47)^, narrative literature review^(24)^.
**Occupational Care for Staff**	Keep the lid down when flushing to dispose of waste. Clothes contaminated with secretions must be bagged and sent to the laundry to be washed separately from the rest^(2,13)^.	Narrative literature review^(2,13)^.
	Appropriate attire during handling and disposal of drain fluids and urine for up to 48 hours post-operatively. Use a waterproof apron, cap, procedure gloves, protective glasses and surgical mask^(1,3,5,13)^.	Experience report^(1)^, case study^(3)^, narrative literature review^(5,13)^.
	Prevent pregnant women or those who intend to become pregnant, people with a history of cancer or immunocompromised individuals from providing assistance to patients undergoing HIPEC, or in the 48 hours postoperatively^(5,7)^.	Narrative literature review^(5,7)^.

Note: ICU – Intensive Care Unit; NPO – nothing by mouth; HIPEC – hyperthermic intraoperative chemotherapy.

Regarding the hemodynamic monitoring category, it is observed that the optimization of individualized hemodynamic status (n = 19) and attention to strict control of fluid balance, with assessment of the volume of content in drains, probes, and tubes (n = 14) were the hemodynamic care with the most citations of studies in the scoping review. Furthermore, the use of an epidural catheter is recommended as the gold standard for analgesia (n = 23), in addition to multimodal analgesia (n = 11), for better control and relief of postoperative pain. Regarding nutritional aspects, it is recommended to start nutrition for the patient as early as possible (n = 14), and in mobilization, to encourage exercise and mobilization on the first postoperative day (n = 9), for patients undergoing cytoreduction and HIPEC during recovery in the ICU.

## DISCUSSION

Based on the mapping carried out of the studies included in the review, 72 care procedures were identified, grouped into 14 areas of care needs for patients undergoing cytoreduction and HIPEC, with postoperative recovery in the ICU. During the intraoperative period, cytoreduction and the use of intra-abdominal chemotherapy triggers important multi-organ changes that may lead the patient to present serious systemic instabilities if there is no rapid intervention by the healthcare team^(7,26,37)^.

Prolonged mechanical ventilation influences the length of stay in the ICU, and has inherent risks, such as ventilator-associated pneumonia (VAP)^(38)^. Extubation is recommended as soon as awakening and ventilatory improvement are observed^(1,28,31,33)^. Early extubation has proven to be an effective strategy for reducing the length of stay in the ICU: in a multicenter study conducted in Japan, including 37,983 patients, hospitals that had a higher proportion of early extubation also had a lower prevalence of respiratory complications, as well as a reduction in hospital costs^(49)^.

After extubation, the patient may benefit from the use of continuous positive airway pressure (CPAP), allowing lung expansion and consequently preventing atelectasis, extubation failures, and the risk of returning to mechanical ventilation^(7,27,28)^. In a study carried out with post-operative lung cancer patients, the use of CPAP, when compared with the routine use of nasal cannula, also promoted less dyspnea and less weight loss one month after surgery^(50)^. Upon admission to the ICU, hemodynamic resuscitation should be performed based on intraoperative performance and status in the first hours of recovery: nursing is responsible for rigorously assessing the hemodynamic pattern, based on the assessment of vital signs of invasive blood pressure, central venous pressure, heart rate, peripheral perfusion and rigorous recording of fluid balance^(13,14,29,37,38)^.

It is expected that in the first 72 hours there will be a loss of approximately four liters of fluids, drained mainly by thoracic and abdominal drains, nasogastric and bladder tubes, which leads to high fluid depletion and consequent risk of hemodynamic instability^(2,26,27)^.

When handling the contents present in drains, the team must take special care regarding the use of personal protective equipment in the first 48 hours in the ICU, to avoid accidental direct contact with the chemotherapy drug still present in the drains and catheters^(1,3,5,13)^. The use of certain chemotherapy drugs during surgery may trigger loss of renal function in the patient in the ICU, requiring rigorous assessment of renal function through creatinine and urea tests, as well as observation of the volume of diuresis eliminated, the ideal being 1 ml/kg/h in the first 72 hours of postoperative hospitalization^(5,13,26,32,36)^.

Postoperative pain management through epidural catheter analgesia was cited by most studies as the most appropriate and safe method for pain control and relief. Adequate pain control implies postoperative recovery with reduction of events, respiratory complications and promotion of mobilization^(24)^. In a study developed with 124 patients in France who underwent cytoreduction and HIPEC, the use of an epidural catheter, associated with multimodal analgesia and a program of early mobilization and respiratory exercises, demonstrated a reduction in the length of ICU stay^(40)^.

Furthermore, a study conducted in Taiwan also demonstrated that adequate pain control is associated with a better quality of life for the patient, as well as an increase in satisfaction with care, representing benefits for both the patient and the institution that serves them^(51)^. Most patients admitted to the ICU will require the use of specific drains.

However, the use of a nasogastric tube should be assessed individually as to its necessity, with guidance for its removal as early as possible^(20)^. Depending on the complexity of surgical manipulation and risk of paralytic ileus, permanence is recommended^(31,33)^. Fasting for more than 24 hours should preferably be avoided in all patients^(4,46)^. A study of 109 patients undergoing cytoreduction and HIPEC recovering in the ICU demonstrated that postoperative fasting longer than 24 hours persisted as a risk factor for prolonged ICU stay and a greater tendency to develop infections^(46)^.

The ERAS guideline recommends starting enteral nutrition as early as possible, still on the first day of admission to the ICU, with progression of the diet modality according to the patient’s acceptance and clinical and surgical condition^(8,19,20,28)^. Early enteral nutrition reduces the risk of complications such as bacterial translocation and malnutrition in critically ill patients^(7,38)^. However, in the presence of postoperative complications such as paralytic ileus, resection of intra-abdominal organs, fistulas and sepsis, the administration of enteral nutrition should be reassessed^(8)^. Given the impossibility of starting enteral nutrition, either orally or via tube, the multidisciplinary team must reassess the administration of total parenteral nutrition, especially in those patients without dietary support for up to three days^(7,8,37,38,44)^.

Infections developed during the ICU recovery period are directly related to morbidity, prolonged hospital stay, and hospital costs^(23)^. Chinese research with 482 patients undergoing cytoreduction and HIPEC demonstrated that the most common sites of infection observed were central venous access (8.1%) and abdominopelvic infection related to the surgical site (5.2%), in addition to being an independent risk factor for perioperative blood loss and ascites^(52)^. Therefore, it is essential that barrier measures against healthcare-related infections be recommended as routine in any ICU, such as adequate hand hygiene for professionals and education for family members and patients, adequate handling of central accesses, measures to prevent ventilator-associated pneumonia (VAP), and adequate maintenance of the closed bladder drainage system^(18,21,33)^.

Nursing must maintain strict glycemic control, aiming at a blood glucose target between 140 and 180 mg/dL^(4,8,19,20,29)^. The development of hyperglycemia is common due to the intense inflammatory process in patients undergoing HIPEC. Therefore, attention should be paid to the need for insulin supplementation^(9,19,26)^. Hypothermia is common upon admission, especially in patients undergoing open surgery. Therefore, strict temperature control is required, with a target above 36°C, and assessment of the need to install heating systems such as thermal blankets or heated fluids^(4,10,14,30,32)^.

Due to the oncological condition, simultaneously with HIPEC, patients may present abnormalities in the coagulation profile, with a predisposition to the formation of thrombi, at risk of developing deep vein thrombosis^(26)^. Pharmacological prophylaxis measures, such as the use of unfractionated heparin by the medical team in the absence of contraindications and bleeding, in association with the use of compression stockings and venous return boots, help to reduce the risk of thrombotic events^(2,5,7,8)^.

In addition to physical care, special attention must be given to the mental health of both the patient and family. It is recommended to involve both, together with the multidisciplinary team, in making therapeutic decisions, as well as providing complete and continuous information about care and possible risks inherent to surgery^(22,47,48)^. Adequate communication among staff, patients and families reduces, especially for the patient, symptoms such as anxiety and stress during the recovery process^(24)^.

Of all the precautions discussed here, most are present in the current ERAS protocol guideline^(8)^. Given the positive postoperative recovery outcomes, the use of interventions discussed in the ERAS protocol provides a reduction in average hospital costs due to the reduction in hospital stay time (7 ± 1.1 days), when compared to their non-use (10 ± 4.5 days), as demonstrated in a study developed in Türkiye^(19)^.

Some limitations are highlighted in this study, such as the fact that most studies are narrative reviews of the literature, consequently presenting a low level of evidence. During the scoping review, the authors observed few studies with more robust methodological designs with precise information on postoperative care in the ICU, which led to the inclusion of reviews to fully verify what was available in the literature on care for this patient profile. Moreover, there were few studies with care directed at nursing assistance, with the theme of generalized care, medicine, or reviews being more present in articles.

There is a clear need to publish studies on nursing care for patients in the postoperative period of cytoreduction and HIPEC with a better quality methodological design and focused on nursing. However, during the evaluation of the studies to be included, a single Brazilian study was observed in the format of a narrative review with an approach to postoperative care in the ICU after cytoreduction and HIPEC. In addition, another Brazilian study addressed only care related to fasting and postoperative nutrition.

Thus, this article is the first scoping review from a nursing perspective, which mapped the main care practices to be performed in the ICU for patients undergoing cytoreduction and HIPEC. The nursing team must know what needs these patients have in the postoperative period and rethink their practices, applying scientific evidence to the care routine, to provide assistance based on the literature, in favor of adequate recovery and safety of the patient and team.

## CONCLUSION

After carrying out the scoping review, it was possible to map 72 care procedures present in the scientific literature for patients undergoing cytoreduction and HIPEC. These care measures were grouped into 14 care areas, to facilitate understanding of the activities performed post-operatively. It was observed that nursing care is similar to that for any patient undergoing a major surgery who recovers in the ICU, with some particularities for the team providing care in the first 48 hours. Of the 42 studies selected, only two were Brazilian articles addressing the topic, one a narrative review, with postoperative care in the ICU, and the other a case study.

Among the mapped care, there was a greater concentration of care related to the themes of hemodynamic and ventilatory monitoring, pain management, nutritional care, and mobilization. The need for studies with more robust methodology regarding scientific evidence of the safety of this care, mainly aimed at the nursing team, was highlighted. Knowing the care practices to be implemented in the ICU for this type of patient allows for safe, scientifically based care that promotes adequate recovery.
