# Supplementary material for: Nursing care for cytoreduction and hyperthermic intraoperative chemotherapy in an Intensive Care Unit: a scoping review
Source: Rev Esc Enferm USP. 2024 Nov 25;58:e20240176. doi: 10.1590/1980-220X-REEUSP-2024-0176en (PMC11593163; doi:10.1590/1980-220X-REEUSP-2024-0176en)
Supplement: Translation

# Translation: Cuidados de enfermagem à citorredução e *hyperthermic intraoperative chemotherapy* em Unidade de Terapia Intensiva: revisão de escopo

**DOI:** 10.1590/1980-220X-REEUSP-2024-0176pt

**Authors:** Aline Branco, Fernanda Cirne Lima Weston, Giovanna da Rosa Soares, Graciele Fernanda da Costa Linch, Rita Catalina Aquino Caregnato

**Affiliations:** 1 Universidade Federal de Ciências da Saúde de Porto Alegre, Porto Alegre, RS, Brasil.

**Keywords:** Quimioterapia Intraperitoneal Hipertérmica, Unidades de Terapia Intensiva, Cuidados Pós-Operatórios, Recuperação Pós-Cirúrgica Melhorada, Cuidados de Enfermagem

## Abstract

**Objetivo:**

Mapear os cuidados de enfermagem pós-operatórios para pacientes críticos adultos e idosos internados em Unidade de Terapia Intensiva após cirurgia de citorredução com quimioterapia intraperitoneal hipertérmica (*hyperthermic intraoperative chemotherapy)*.

**Método:**

Revisão de escopo segundo metodologia do JBI, com artigos extraídos de bases de dados e literatura cinzenta, sem delimitação de idioma ou data de publicação. Realizou-se o processo de seleção dos estudos e extração dos resultados por dois revisores independentes, por meio dos programas *EndNote* ^®^ e *Rayyan* ^®^. Utilizou-se o PRISMA *Extension for Scoping Reviews* para a redação, registrando-se na *Open Science Framework*.

**Resultados:**

Selecionados 42 estudos. Análise evidenciou 72 cuidados agrupados em 14 áreas assistenciais. A utilização de cateter epidural para analgesia, otimização do status hemodinâmico individualizado, e controle rigoroso do balanço hídrico foram os cuidados mais citados.

**Conclusão:**

O mapeamento identificou cuidados de enfermagem pós-operatórios semelhantes às cirurgias de grande porte de pacientes recuperados na Unidade de Terapia Intensiva, com indicação de uso de equipamentos de proteção individual para os profissionais ao manipular drenos nas primeiras 48 horas de admissão.

## INTRODUÇÃO

Nas últimas décadas, observa-se aumento na incidência de diversas neoplasias, exigindo constante busca por novas modalidades de tratamentos oncológicos. Dentre as abordagens cirúrgicas, destaca-se a citorredução associada à quimioterapia intraperitoneal hipertérmica (*hyperthermic intraoperative chemotherapy,* HIPEC), desenvolvida na década de 80 e indicada para a remoção de tumores malignos abdominais em órgãos reprodutores, gastrintestinais e de peritônio^(1, 2, 3)^.

A citorredução é caracterizada pela retirada de sítios tumorais e perilesionais visíveis de forma manual pelo cirurgião; enquanto isso, a HIPEC, comumente realizada no mesmo momento intraoperatório, consiste na aplicação de quimioterápico com temperatura de 40 a 43º na cavidade abdominal, sendo mantido no local por 60 a 120 minutos, a fim de permitir sua ação direta nos órgãos acometidos pela neoplasia^(4,5)^. A alta temperatura da quimioterapia é essencial, pois estimula a resposta imunológica, a ação direta do fármaco sobre as áreas neoplásicas e a apoptose de células tumorais no organismo^(6,7)^.

A associação de ambos os procedimentos resulta em uma cirurgia de alta complexidade, portanto, os pacientes são encaminhados para a Unidade de Terapia Intensiva (UTI) para a recuperação pós-operatória. A eficácia do tratamento não depende somente da aplicação da técnica cirúrgica correta, mas também do adequado manejo pós-operatório por parte da equipe multidisciplinar. Atualmente, as recomendações internacionais *Enhanced Recovery After Surgery* (ERAS) específicas à citorredução associada a HIPEC apresentam uma seção destinada exclusivamente aos cuidados pós-operatórios^(8,9)^. O ERAS tem por objetivo trazer pontos cruciais de cuidado que devem ser implementados aos pacientes desde o primeiro dia do pós-operatório, a fim de tornar o cuidado mais eficiente e avançar à recuperação plena dos pacientes^(9)^.

Dentre as principais práticas recomendadas pelo ERAS, estão a remoção de cateter urinário até o 3° dia de pós-operatório, analgesia por cateter epidural, mobilização precoce e introdução de líquidos claros no primeiro dia pós-operatório^(8)^, cuidados que, dentro da rotina assistencial da UTI, são realizados pela equipe multiprofissional. A enfermagem na UTI, integrante da equipe multiprofissional, presta a assistência à beira leito de forma contínua e assume a responsabilidade de uma parte significativa dos cuidados pós-operatórios. No escopo desses cuidados, são incluídos o manejo álgico adequado, preferencialmente por meio da analgesia epidural, baseado em constantes avaliações da dor; a administração de antieméticos; a mobilização precoce; constante monitoramento glicêmico e térmico; realização de adequado balanço hídrico; cuidados com sondas e drenos^(8,9,10)^.

Apesar de ser reconhecida a importância dos cuidados de enfermagem pós-operatórios em UTI para o sucesso terapêutico da citorredução associada à HIPEC, ainda são escassos os estudos que os abordam. A atuação da enfermagem no intraoperatório na assistência ao paciente durante a cirurgia, pela manipulação dos dispositivos de infusão do quimioterápico, auxílio nos cuidados anestésicos e proteção dos profissionais durante a fase de HIPEC, é abordada na literatura^(11,12)^. Evidenciou-se um estudo brasileiro sobre cuidados pós-operatórios para pacientes submetidos à HIPEC, porém no formato de revisão narrativa da literatura^(13)^.

As pesquisas majoritariamente enfatizam as condutas médicas, representando uma barreira no estabelecimento de protocolos assistenciais que beneficiem esses pacientes^(8,9,14)^. Também não foram identificados protocolos e diretrizes específicos à equipe de enfermagem, o que pode dificultar uma uniformização do cuidado e a concretização de uma prática baseada em evidências na UTI^(13)^. Portanto, identifica-se uma lacuna no conhecimento na enfermagem sobre a assistência pós-operatória em pacientes submetidos à citorredução associada à HIPEC. Desta forma, decidiu-se realizar um mapeamento da literatura a fim de identificar quais cuidados pós-operatórios são preconizados para esses pacientes de acordo com a literatura científica. Este artigo tem por objetivo mapear os cuidados de enfermagem pós-operatórios para pacientes críticos adultos e idosos internados em UTI após cirurgia de citorredução com HIPEC.

## MÉTODO

### Desenho Do Estudo

Realizou-se uma revisão de escopo por ser o método indicado para o mapeamento de evidências, a fim de identificar o estado da arte sobre conceitos e teorias, evidências disponíveis e levantar lacunas do conhecimento^(15)^. Para o desenvolvimento desta revisão, seguiram-se as diretrizes da JBI, a partir destas etapas: identificação da questão de pesquisa; verificação dos estudos relevantes nas fontes de evidências; seleção dos estudos a incluir na revisão conforme critérios de elegibilidade; mapeamento e extração dos dados dos estudos incluídos na revisão; e organização e apresentação dos resultados^(16)^. Para garantia da qualidade e transparência da redação utilizou-se o PRISMA *Extension for Scoping Reviews* (PRISMA-ScR) com análise se todos os itens de desenvolvimento da revisão foram contemplados^(17)^. Uma pesquisa preliminar na PROSPERO, MEDLINE, *Cochrane Database of Systematic Reviews*, e JBI *Evidence Synthesis* foi realizada, e não se evidenciaram protocolos, revisões sistemáticas ou revisões de escopo em andamento sobre o tema. Elaborou-se um protocolo para revisão de escopo*,* registrado na *Open Science Framework* (OSF) sob DOI 10.17605/OSF.IO/UZH5K.

### Coleta De Dados

Na primeira etapa, estruturou-se a questão de pesquisa pelo mnemônico PCC^(15,16)^ onde: (P)opulação refere-se a pacientes oncológicos adultos e idosos submetidos ao procedimento cirúrgico de citorredução com HIPEC que realizem a recuperação na UTI; (C)ontexto, estudos cujo contexto seja exclusivamente o ambiente da Unidade de Terapia Intensiva e; (C)onceito, cuidados de enfermagem pós-operatórios necessários para a recuperação destes pacientes, gerando a pergunta “quais os cuidados de enfermagem pós-operatórios indicados aos pacientes críticos adultos e idosos internados em UTI após cirurgia de citorredução com HIPEC?”.

A estratégia de pesquisa procedeu-se em três tempos. Inicialmente, buscou-se na PubMed (MEDLINE) estudos acerca de cuidados pós-operatórios de citorredução e HIPEC, para verificar quais os termos mais comumente utilizados. Procedeu-se para a busca destes termos sob o formato de descritores indexados em português nos Descritores em Ciências da Saúde (DeCS) e em inglês no *Medical Subject Headings (MeSH).* Após a seleção dos descritores indexados, elaboraram-se duas estratégias de pesquisa iniciais para a PubMed (MEDLINE) e CINAHL, a fim de verificar se estas contemplavam estudos que respondessem ao objetivo e questão de pesquisa, com busca realizada em 5 de agosto de 2023 retornando 372 artigos, conforme apresenta o Quadro 1. As estratégias desenvolvidas para as demais bases de dados podem ser consultadas na íntegra no protocolo de revisão de escopo registrado na OSF.

**Quadro 1:** Estratégias de buscas para as bases de dados PubMed (MEDLINE) e CINAHL - Porto Alegre, RS, Brasil, 2024.

Base de dados	Estratégias de buscas	Resultados
**PubMed (MEDLINE)**	(Cytoreduction Surgical Procedures[mh] OR Cytoreduct*[tiab] OR Debulk*[tiab]) AND (Hyperthermic Intraperitoneal Chemotherapy[mh] OR Hyperthermia, Induced[mh:noexp] OR Hyperthermic Intraperitoneal Chemotherap*[tiab] OR HIPEC[tiab] OR Hot Chemotherap*[tiab] OR Intraperitoneal Hyperthermic Chemotherap*[tiab] OR Induced Hypertherm*[tiab] OR Therapeutic Hypertherm*[tiab] OR Thermotherap*[tiab] OR Fever Therap*[tiab] OR Local Hypertherm*[tiab]) AND (Intensive Care Units[mh:noexp] OR Respiratory Care Units[mh] OR Critical Care[mh] OR Critical Care Nursing[mh] OR Intensive care*[tiab] OR Critical care*[tiab] OR ICU[tiab] OR Care Unit*[tiab] OR Nursing Care[mh:noexp] OR Medical-Surgical Nursing[mh] OR Oncology Nursing[mh] OR Nurses[mh] OR Nursing[tiab] OR Nurse*[tiab] OR Postoperative Period[mh] OR Postoperative Care[mh] OR Recovery Room[mh] OR Enhanced Recovery After Surgery[mh] OR Postoperat*[ti] OR Post-operat*[ti] OR Post-surg*[ti] OR Recover*[ti])	315
**CINAHL**	(MH “Cytoreduction Surgical Procedures” OR TI ( Cytoreduct* OR Debulk* ) OR AB ( Cytoreduct* OR Debulk* ) OR SU ( Cytoreduct* OR Debulk* ) AND (MH ( “Hyperthermic Intraperitoneal Chemotherapy” OR “Hyperthermia, Induced” ) OR TI ( “Hyperthermic Intraperitoneal Chemotherap*” OR “HIPEC” OR “Hot Chemotherap*” OR “Intraperitoneal Hyperthermic Chemotherap*” OR “Induced Hypertherm*” OR “Therapeutic Hypertherm*” OR “Thermotherap*” OR “Fever Therap*” OR “Local Hypertherm*” ) OR AB ( “Hyperthermic Intraperitoneal Chemotherap*” OR “HIPEC” OR “Hot Chemotherap*” OR “Intraperitoneal Hyperthermic Chemotherap*” OR “Induced Hypertherm*” OR “Therapeutic Hypertherm*” OR “Thermotherap*” OR “Fever Therap*” OR “Local Hypertherm*” ) OR SU ( “Hyperthermic Intraperitoneal Chemotherap*” OR “HIPEC” OR “Hot Chemotherap*” OR “Intraperitoneal Hyperthermic Chemotherap*” OR “Induced Hypertherm*” OR “Therapeutic Hypertherm*” OR “Thermotherap*” OR “Fever Therap*” OR “Local Hypertherm*” ) AND (MH ( “Intensive Care Units” OR “Respiratory Care Units” OR “Critical Care” OR “Critical Care Nursing” OR “Nursing Care” OR “Medical-Surgical Nursing” OR “Oncology Nursing” OR “Nurses” OR “Postoperative Period” OR “Postoperative Care” OR “Recovery Room” OR “Enhanced Recovery After Surgery” ) OR TI ( “Intensive care*” OR “Critical care*” OR “ICU” OR “Care Unit*” OR “Nursing” OR “Nurse*” OR “Postoperat*” OR “Post-operat*” OR Post-surg* OR Recover* ) OR AB ( “Intensive care*” OR “Critical care*” OR “ICU” OR “Care Unit*” OR “Nursing” OR “Nurse*” ) OR SU ( “Intensive care*” OR “Critical care*” OR “ICU” OR “Care Unit*” OR “Nursing” OR “Nurse*” )	57

Nota: Pubmed - *National Library of Medicine*; CINAHL - *Cumulative Index to Nursing and Allied Health Literature*.

As estratégias iniciais foram posteriormente adaptadas para as bases de dados Biblioteca Virtual em Saúde (BVS), Scopus (Elsevier), Embase (Elsevier), CINAHL (Ebsco), CENTRAL (*John Wiley*) e *Web of Science* (*Clarivate Analytics*). O acesso às bases Scopus, Embase, CENTRAL e *Web of Science* ocorreu pelo Portal de Periódicos, da Coordenação de Aperfeiçoamento de Pessoal de Nível Superior (CAPES) do Brasil, enquanto a CINAHL por meio da Ebsco, com busca dos artigos em todas as bases de dados, de 26 a 30 de Agosto de 2023. Ainda, consultaram-se as listas de referências dos artigos incluídos na revisão de escopo para identificar estudos relevantes, foram realizadas buscas por teses e dissertações na base ProQuest, e literatura cinzenta na *System for Information on Grey Literature in Europe, British Library EThOS* e *Centre for Reviews and Dissemination* (*York University*). A busca por descritores e elaboração final das estratégias foram realizadas por bibliotecário especialista em pesquisas na área da saúde. Todo o processo de seleção dos estudos incluídos na revisão até a extração de dados ocorreu de forma blindada por duas pesquisadoras, e em sinal de divergência dos estudos incluídos ou resultados extraídos, acionou-se uma terceira revisora.

### Critérios de Elegibilidade

Como fontes de informação, elegeram-se estudos primários de metodologia qualitativa e quantitativa, estudos de revisão, teses, dissertações e capítulos de livro disponíveis na íntegra em meio eletrônico. Excluíram-se cartas, artigos de opinião, editoriais e resumos científicos, documentos duplicados, além de estudos que contemplavam os períodos intra e transoperatório e que tinham como público crianças e adolescentes. Não houve restrições quanto ao ano de publicação ou idioma. Optou-se por não restringir período, pois na elaboração das primeiras estratégias de busca observaram-se poucos estudos abordando a temática. A seleção inicial dos estudos ocorreu a partir da exclusão das duplicatas, procedendo à leitura de título e resumo. Uma vez contemplando o tema e pergunta de pesquisa, seguiu-se para a leitura na íntegra. Os artigos foram inicialmente exportados para o *EndNote* ^®^ onde procedeu-se a remoção de duplicatas, e posteriormente a seleção por meio do *Rayyan* ^®^. Para aqueles lidos na íntegra, dois pesquisadores de forma blindada recomendaram a inclusão e exclusão, e em divergência acionou-se um terceiro revisor.

### Extração e Análise Dos Dados

Os autores elaboraram um instrumento próprio baseado no recomendado pelo JBI para a extração dos dados dos estudos contemplados para a revisão de escopo*,* composto por título, autor, ano, revista, país, objetivo da pesquisa, desenho metodológico, população e cuidados com pacientes em UTI submetidos à citorredução associada à HIPEC. Dentre os cuidados, extraíram-se informações referentes à monitorização hemodinâmica e ventilatória, avaliação e assistência à dor do paciente, padrão neurológico, cuidados com feridas operatórias, drenos, sondas, ostomia, tubos e cateteres, prevenção e manejo racional de infecções relacionada à assistência à saúde (IRAS), controle glicêmico, térmico e nutricional, mobilização, cuidados com profilaxia de úlcera de estresse e trombose venosa profunda, cuidados com saúde mental e atenção à família, e cuidados ocupacionais com a equipe à exposição do quimioterápico. Essas áreas assistenciais a serem exploradas, quantos aos cuidados mapeados respectivos, foram determinadas devido à experiência prévia da autora principal que presta cuidado aos pacientes submetidos à citorredução e HIPEC na UTI.

Os dados foram documentados em planilha Excel^®^ para melhor extração dos resultados e avaliação dos dados. Realizou-se a análise descritiva em percentual de frequência da citação de determinado cuidado, bem como o perfil das publicações. Posteriormente os resultados mapeados foram agrupados conforme objeto assistencial de cuidado.

### Aspectos Éticos

Por tratar-se de um estudo de revisão de escopo com a utilização de dados secundários disponíveis publicamente, dispensa-se aprovação em Comitê de Ética e Pesquisa. Foram respeitados os critérios de autoria dos autores e veracidade das informações.

## RESULTADOS

Durante o processo de seleção dos estudos, retornaram inicialmente como resultados 1.758 artigos. Após a exclusão dos estudos duplicados e aplicação dos critérios de elegibilidade, resultaram ao todo 42 estudos, sendo 40 artigos científicos e dois capítulos de livro incluídos na revisão de escopo, conforme demonstrado na figura 1.

**Figura 1:** Fluxograma PRISMA do processo de seleção dos artigos. Porto Alegre, RS, Brasil, 2024.

Nota: Pubmed – *National Library of Medicine*; CINAHL – *Cumulative Index to Nursing and Allied Health Literature*; BVS – Biblioteca Virtual em Saúde.

O período de publicação estendeu-se de 2008 a 2022, sendo o ano de 2019^(4,14,18,19,20,21^ o mais prevalente de publicação. A maior concentração de publicações foi observada na revista *European Journal of Surgical Oncology* ^(7,8,23,24,25)^, com autorias concentrando-se com maior prevalência nos Estados Unidos (n = 14), Índia (n = 5), Alemanha (n = 4), Itália (n = 3), China (n = 3), Suécia (n = 3), Brasil (n = 2), Espanha (n = 2), e um artigo nos seguintes países: França, Turquia, Inglaterra, Dinamarca, Polônia e Irlanda. Ainda, a grande maioria dos resultados compôs revisão narrativa da literatura (n = 16), seguidos dos estudos observacionais transversais (n = 8) e estudo de coorte retrospectiva (n = 8), revisão sistemática (n = 3), estudos qualitativos (n = 3), capítulos de livro (n = 2), estudos de caso (n = 1) e relato de experiência (n = 1).

O maior motivo de exclusão da amostra foi a metodologia inadequada (53%), referindo-se a artigos que se apresentavam sob o formato de resumos científicos publicados em anais. Com relação às bases de dados dos 42 resultados incluídos na revisão, a PubMed (60%) concentrou a maioria dos estudos, seguida de SCOPUS e *Web of Science* (10%), listas de referências (21%), Embase (7%) e CINAHL (2%). Com o objetivo de mapear os cuidados pós-operatórios de enfermagem a serem realizados em pacientes submetidos à citorredução e HIPEC com recuperação na UTI, os autores decidiram por caracterizar os cuidados em áreas específicas de necessidades de assistenciais ao paciente no pós-operatório. Por meio da aplicação de instrumento próprio de extração de dados, foram observados inicialmente 177 cuidados abordados individualmente pelos estudos.

Conforme os cuidados foram se repetindo nos estudos incluídos na revisão de escopo, realizou-se um agrupamento desses nas suas áreas assistenciais, referenciando seus respectivos estudos citados. Assim, das necessidades assistenciais inicialmente mapeadas chegou-se ao resultado final de 72 cuidados. Os cuidados de enfermagem mapeados no pós-operatório em UTI para pacientes submetidos à citorredução e HIPEC podem ser observados no Quadro 2.

**Quadro 2:** Áreas assistenciais e seus respectivos cuidados de enfermagem para pacientes submetidos à citorredução e HIPEC com recuperação pós-operatória em UTI – Porto Alegre, RS, Brasil, 2024.

Áreas Assistenciais	Cuidados mapeados	Apresentação dos estudos
**Monitorização Hemodinâmica**	Observar a necessidade de reposição de fluidos, transfusão de hemoderivados, eletrólitos e início de inotrópicos ou vasopressores conforme a avaliação hemodinâmica individualizada^(1,2,4,5,7,9,10,14,26,27,28,29,30,31,32,33,34,35,36)^.	Revisão sistemática^(4,9)^, estudo transversal^(10,28,31,35)^, revisão narrativa da literatura^(2,5,7,14,26,27,29,32,34,36)^, relato de experiência^(1)^, capítulo de livro^(30,33)^.
	Controlar rigorosamente o balanço hídrico, monitorando ganhos e perdas de líquidos por drenos torácicos, abdominais, sondas nasogástricas/nasoenterais, e sonda vesical/diurese espontânea^(1,2,4,5,7,13,27,29,30,32,33,34,36,37)^.	Revisão sistemática^(4)^, revisão narrativa da literatura^(2,5,7,13,27,29,32,34,36,37)^, relato de experiência^(1)^, capítulo de livro^(30,33)^.
	Avaliar os exames laboratoriais: observando os valores dos eletrólitos, perfil de coagulação, hematócrito e hemoglobina, equilíbrio ácido-base, lactato e demais exames pertinentes^(2,4,5,13,14,29,30,36,37,38)^.	Revisão sistemática^(4)^, estudo transversal^(38)^, revisão narrativa da literatura^(2,5,13,14,29,30,36,37)^.
	Monitorar sinais de sangramento e hemorragia^(3,5,7,29,30)^.	Revisão narrativa da literatura^(5,7,29)^, estudo de caso^(3)^, capítulo de livro^(30)^.
	Monitorar os sinais vitais e padrão hemodinâmico, a partir da avaliação de pressão arterial média invasiva, pressão venosa central, débito e índice cardíaco e frequência cardíaca, além da perfusão periférica^(1,13,14,29,33,37,38)^.	Estudo transversal^(38)^, relato de experiência^(1)^, revisão narrativa da literatura^(13,14,29,37)^, capítulo de livro^(33)^.
	Atentar para função renal, observando volume urinário e avaliação de exames laboratoriais de ureia e creatinina, observação de sinais de edema, aferição do peso corporal e exames de imagem^(1,5,7,9,13,26,28,30,32,36)^.	Revisão sistemática^(9)^, estudo transversal^(28)^, relato de experiência^(1)^, revisão narrativa da literatura^(5,7,13,26,32,36)^, capítulo de livro^(30)^.
	Observar necessidade de monitorização da pressão intra-abdominal, principalmente para pacientes que apresentaram pressão acima de 12 cmH2O durante a cirurgia^(39)^.	Estudo transversal^(39)^.
**Ventilação Mecânica e Oxigenoterapia**	Avaliar necessidade de manter paciente em ventilação mecânica invasiva na UTI, considerando a instabilidade hemodinâmica e complicações relacionadas ao intraoperatório e pós-operatório^(2,4,13,14,26,29,32,37,38)^.	Revisão sistemática^(4)^, estudo transversal^(38)^, revisão narrativa da literatura^(2,13,14,26,29,32,37)^.
	Optar pelo desmame e extubação precoce tão logo quanto possível na sala cirúrgica ou UTI^(1,2,4,14,28,29,31,32,33)^.	Revisão sistemática^(4)^, estudo transversal^(28,31)^, relato de experiência^(1)^, revisão narrativa da literatura^(2,14,29,32)^, capítulo de livro^(33)^.
	Optar, após extubação, pela ventilação não invasiva como pressão positiva contínua (CPAP) ou cânula nasal de alto fluxo (CNAF), com a equipe multidisciplinar^(4,7,25,27,29,32,33)^.	Revisão sistemática^(4)^, estudo de coorte retrospectivo^(25)^, revisão narrativa da literatura^(7,27,29,32)^, capítulo de livro^(33)^.
	Realizar higiene brônquica por meio da aspiração de secreções das vias aéreas e do estímulo à tosse, juntamente à exercícios de respiração profunda e espirometria^(5,9,13,33,40)^.	Revisão sistemática^(9)^, estudo de coorte retrospectivo^(40)^, revisão narrativa da literatura^(5,13)^, capítulo de livro^(33)^.
	Observar sinais de complicações ventilatórias no pós-operatório^(13,26,31,32,37,38)^.	Estudo transversal^(31,38)^, revisão narrativa da literatura^(13,26,32,37)^.
	Utilizar sonda nasogástrica para descompressão e evitar risco de aspirações^(37)^.	Revisão narrativa da literatura^(37)^.
	Monitorar padrão respiratório, com ausculta pulmonar e documentação dos sons a cada 4 horas^(1,2,13,28,31,33,36,37,38)^.	Estudo transversal^(28,31,38)^, relato de experiência^(1)^, revisão narrativa da literatura^(2,13,36,37)^, capítulo de livro^(33)^.
**Padrão Neurológico**	Monitorar o processo de despertar do paciente^(41)^.	Estudo observacional transversal^(41)^.
**Avaliação e Assistência à Dor do Paciente `**	Utilizar analgesia multimodal: combinação de anestésicos locais ou opioides no cateter epidural com anti-inflamatórios não-esteroidais ou demais analgésicos^(2,8,9,10,14,18,19,20,25,40,42)^.	Revisão sistemática^(8,9)^, estudo de coorte^(19,25,40,42)^, estudo transversal^(10,20)^, revisão narrativa da literatura^(2,14,18)^.
	Poupar o uso de opioides endovenosos a partir do uso de outras modalidades de analgesia como o cateter epidural^(8,9,10,27,28)^.	Revisão sistemática^(8,9)^, estudo transversal^(10,28)^, revisão narrativa da literatura^(27)^.
	Estabelecer serviços especializados em dor aguda, na modalidade “Serviço de Dor”. Em casos de dor crônica, acionar a equipe de Cuidados Paliativos^(26,32,42)^.	Estudo de coorte^(42)^, revisão narrativa da literatura^(26,32)^.
	Realizar analgesia por meio de cateter epidural como método seguro para controle da dor^(4,7,8,9,14,18,19,20,24,25,26,27,29,31,32,33,34,36,38,40,41,42,43)^.	Revisão sistemática^(4,8,9)^, estudo de coorte^(19,25,40,42)^, estudo transversal^(20,31,38,41)^, revisão narrativa da literatura^(7,14,18,24,26,27,29,32,34,36,43)^, capítulo de livro^(33)^.
	Manter o cateter epidural em uso até a transição da analgesia para via oral ou IV e descontinuidade do cateter^(4,8,13,19,24-26,36,40,42)^.	Revisão sistemática^(4,8)^, estudo de coorte^(19,25,40,42)^, revisão narrativa da literatura^(13,24,26,36)^.
	Manter a avaliação e cuidados para alívio da dor, sendo documentada a cada 4 horas com a utilização de escalas apropriadas^(3,13,33)^.	Estudo de caso^(3)^, revisão narrativa da literatura^(13)^, capítulo de livro^(33)^.
	Manejar a dor por meio da Analgesia Controlada pelo Paciente (PCA) ou bloqueio de planos transversos pelo anestesista (TAP), sendo associados à analgesia multimodal^(10,13,14,18,25,33,36,42,43)^.	Estudo de coorte^(25,42)^, estudo transversal^(10)^, revisão narrativa da literatura^(13,14,18,36,43)^.
	Utilizar a PCA somente com o paciente acordado e instruído sobre seu uso^(13,33)^.	Revisão narrativa da literatura^(13)^, capítulo de livro^(33)^.
	Manter cateter epidural em uso para alívio da dor entre 72 a 96 horas, alguns estudos sugerindo o uso até o 5° dia e 8° dia de pós-operatório^(4,8,13,14,24,25,26,36)^.	Revisão sistemática^(4,8)^, estudo de coorte^(25)^, revisão narrativa da literatura^(13,14,24,26,36)^.
	Controlar a infusão analgésica via cateter epidural conforme padrão hemodinâmico e neurológico. Manter ambiente asséptico no manuseio e manutenção^(8,26,42)^.	Revisão sistemática^(8)^, estudo de coorte^(42)^, revisão narrativa da literatura^(26)^.
**Ferida Operatória**	Avaliar a incisão cirúrgica para sinais de infecção mantendo protegida com curativo estéril e observar sangramento ou presença de fístulas^(5,33)^.	Revisão narrativa da literatura^(5)^, capítulo de livro^(33)^.
	Para prevenir deiscência, orientar paciente a tossir utilizando um travesseiro pressionando firmemente a incisão^(5,33)^.	Revisão narrativa da literatura^(5)^, capítulo de livro^(33)^.
	Avaliar curativo de pressão negativa quanto a integridade e funcionamento^(36)^.	Revisão narrativa da literatura^(36)^.
**Cuidados com Drenos, Ostomias, Sondas, Tubos e Cateteres**	Avaliar volume e aspecto dos fluídos drenados em drenos torácicos, abdominais e sondas nasoenterais ou nasogástricas^(2,5,13,26,33,37)^.	Revisão narrativa da literatura^(2,5,13,26,37)^, capítulo de livro^(33)^.
	Observar aspecto de secreção de ostomas, ferida operatória e reto. Comunicar equipe médica se houver sinais de alteração de conteúdo^(13,37)^.	Revisão narrativa da literatura^(13,37)^.
	Utilizar gazes estéreis secas para proteção da pele em torno dos drenos^(33,36)^.	Revisão narrativa da literatura^(36)^, capítulo de livro^(33)^.
	Avaliar diariamente a necessidade de manter sonda nasogástrica e drenagem de conteúdo residual^(10,20)^.	Estudo transversal^(10,20)^.
	Observar volume dos drenos torácicos e necessidade de permanência. Os reservatórios nunca deverão atingir mais da metade, monitorando o volume a cada 1-2 horas^(13,33)^.	Revisão narrativa da literatura^(13)^, capítulo de livro^(33)^.
	Atentar que não é recomendada a instalação de sonda nasogástrica para todos os pacientes, se não houver riscos de atraso para esvaziamento gástrico por ressecção de omento^(8,28)^.	Revisão sistemática^(8)^, estudo transversal^(28)^.
	Retirar cateter urinário, sonda nasogástrica, drenos torácicos e abdominais em até 72 horas de internação, ou tão logo quanto possível^(10,19,20,28)^.	Estudo de coorte^(19)^, estudo transversal^(10,20,28)^.
	Introduzir o paciente e família nos cuidados com a ostomia^(18)^.	Revisão narrativa da literatura^(18)^.
**Prevenção e Manejo Racional de Infecções**	Higienizar adequadamente as mãos com água e sabão principalmente antes e após contato com drenos, secreções e sítios cirúrgicos do paciente^(18,33)^.	Revisão narrativa da literatura^(18)^, capítulo de livro^(33)^.
	Envolver família e paciente na educação dos sinais e sintomas de infecção de sítio cirúrgico, e instruir sobre a lavagem das mãos e uso de equipamento de proteção pessoal^(18)^.	Revisão narrativa da literatura^(18)^.
	Manter ambiente asséptico à inserção de cateter centrais, utilizando ultrassom Doppler para guiar a inserção, bem como adequada manipulação das vias e higiene das mãos^(21,37)^.	Estudo de coorte^(21)^, revisão narrativa da literatura^(37)^.
	Usar antibióticos de forma racional e escalonamento conforme resultados de culturas apropriadas e conforme o quadro do paciente^(2,4,29)^.	Revisão sistemática^(4)^, revisão narrativa da literatura^(2,29)^.
	Atentar para sinais de infecção pela alteração da composição das drenagens em drenos e da ferida operatória^(5,23,37)^.	Estudo de coorte^(23)^, revisão narrativa da literatura^(5,37)^.
**Controle Glicêmico**	Manter controle glicêmico rigoroso. O alvo do valor de glicose em pacientes críticos deve estar entre 140 – 180 mg/dL^(4,8,9,19,26,29)^.	Revisão sistemática^(4,8,9)^, estudo de coorte^(19)^, revisão narrativa da literatura^(26,29)^.
	Atentar para a hiperglicemia, comumente observada, necessitando do uso de insulinas, principalmente em pacientes submetidos a intervenções no pâncreas^(4,8,19,26)^.	Revisão sistemática^(4,8)^, estudo de coorte^(19)^, revisão narrativa da literatura^(26)^.
	Avaliar a necessidade de infusão contínua de insulina para correção de hiperglicemia^(9,29)^.	Revisão sistemática^(9)^, revisão narrativa da literatura^(29)^.
**Controle Térmico**	Monitorizar a temperatura no pós-operatório, contínua e rigorosamente, por meio de fluidos aquecidos e mantas térmicas^(10,14,32)^.	Estudo transversal^(10)^, revisão narrativa da literatura^(14,32)^.
	Manter a normotermia (temperatura corporal > 36°C) e avaliar o lactato sérico junto aos outros marcadores inflamatórios para avaliar perfusão tecidual^(4,10,30,32)^.	Revisão sistemática^(4)^, estudo transversal^(10)^, revisão narrativa da literatura^(32)^, capítulo de livro^(30)^.
**Controle Nutricional**	Dar preferência para a nutrição oral. Se houver complicações abdominais nas quais o paciente não tolere nutrição entérica, iniciar por via parenteral se o atraso nutritivo superar mais de três dias^(4,7,9,14,18,36,37,38,44,45)^.	Revisão sistemática^(4,9)^, estudo de coorte^(44)^, estudo transversal^(38)^, revisão narrativa da literatura^(7,14,18,36,37,45)^.
	Iniciar a nutrição o mais cedo possível, ofertando líquidos claros no primeiro dia pós-cirúrgico. Progredir conforme aceitação, tolerância e avaliação clínica para dieta com baixo teor de resíduos, e após para alimentos sólidos^(7,8,9,10,14,19,20,25,28,29,32,33,38,45)^.	Revisão sistemática^(8,9)^, estudo de coorte^(19,25)^, estudo transversal^(10,20,28,38)^, revisão narrativa da literatura^(7,14,29,32,45)^, capítulo de livro^(33)^.
	Avaliar as funções intestinais pela ausculta de ruídos hidroaéreos, pela flatulência, evacuação e pelo volume de resíduo gástrico, documentando aceitação e ganho de peso^(8,9,13,24,28,33,36)^.	Revisão sistemática^(8,9)^, estudo transversal^(28)^, revisão narrativa da literatura^(13,24,36)^, capítulo de livro^(33)^.
	Observar a tolerância do paciente à modalidade nutricional oferecida para identificar aqueles com nutrição insuficiente e sinais de complicações^(18,24,33)^.	Revisão narrativa da literatura^(18,24)^, capítulo de livro^(33)^.
	Associar oferta de fluidos endovenosos para aporte energético, caso seja necessário ^(4,7,13,14,24,28,36,37,38)^.	Revisão sistemática^(4)^, estudo transversal^(28,38)^, revisão narrativa da literatura^(7,13,14,24,36,37)^.
	Iniciar nutrição parenteral total (NPT) o mais precoce possível em sinais de complicações que impeçam via entérica. A duração da NPT é baseada no status nutricional, complicações e contagem calórica^(4,7,30,36,37,38)^.	Revisão sistemática^(4)^, estudo transversal^(38)^, revisão narrativa da literatura^(7,36,37)^, capítulo de livro^(30)^.
	Evitar o jejum pós-operatório indevido acima de 24 horas, por ser um fator de risco para internação prolongada na UTI^(7,37,38,46)^.	Estudo de coorte^(46)^, estudo transversal^(38)^, revisão narrativa da literatura^(7,37)^.
	Não administrar alimentação via oral (NPO), se risco devido à condição cirúrgica, até o retorno dos ruídos hidroaéreos, flatulência e diminuição dos resíduos gástricos^(13,33)^.	Revisão narrativa da literatura^(13)^, capítulo de livro^(33)^.
	Utilizar pró-cinéticos, laxativos e gomas de mascar para a prevenção de íleo paralítico^(8,19,28)^.	Revisão sistemática^(8)^, estudo de coorte^(19)^, estudo transversal^(28)^.
**Mobilização**	Estimular a mobilização precoce desde o primeiro dia pós-operatório^(4,5,8,9,10,20,24,28,40)^.	Revisão sistemática^(4,8,9)^, estudo de coorte^(40)^, estudo transversal^(10,20,28)^, revisão narrativa da literatura^(5,24,40)^.
	Realizar mudanças de decúbito no leito a cada duas horas, com cuidado aos drenos torácicos e abdominais^(13,33)^.	Revisão narrativa da literatura^(13)^, capítulo de livro^(33)^.
	Mobilizar no leito já no primeiro dia pós-operatório e entre o primeiro e quarto dia pós-operatório encorajar a saída da cama para poltrona e deambulação^(7,8,9,10,19,24,25,33,40)^.	Revisão sistemática^(8,9)^, estudo de coorte^(19,25,40)^, estudo transversal^(10)^, revisão narrativa da literatura^(7,24)^, capítulo de livro^(33)^.
	Monitorar drenagem e preservação de drenos, incisão cirúrgica e mudanças hemodinâmicas durante a mobilização^(38,41)^.	Estudo transversal^(38,41)^.
**Profilaxia Úlcera de Estresse e TVP**	Prevenir úlcera de estresse, utilizando antagonista de receptor H2 e inibidores da bomba de prótons, conforme a prescrição médica^(2,29,31)^.	Estudo transversal^(31)^, revisão narrativa da literatura^(2,29)^.
	Administrar, conforme prescrição médica, profilaxia farmacológica com heparina de baixo peso molecular ou heparina não-fracionada, e usar compressores de membros inferiores intermitentes e meias elásticas de compressão graduada^(2,4,5,7,8,9,14,19,29,33)^.	Revisão sistemática^(4,8,9)^, estudo de coorte^(19)^, revisão narrativa da literatura^(2,5,7,14,29)^, capítulo de livro^(33)^.
	Iniciar a profilaxia farmacológica 12 horas antes da cirurgia, estendendo-se até a quarta semana de internação hospitalar ou até à completa mobilização^(4,8,9,19)^.	Revisão sistemática^(4,8,9)^, estudo de coorte^(19)^.
	Promover a adequada hidratação, deambulação precoce, exercícios de pernas enquanto estiver na cama ou na cadeira^(33)^.	Capítulo de livro^(33)^.
**Saúde Mental e Atenção à Família**	Ajustar dimensionamento de enfermagem para melhor cuidado a este perfil de pacientes^(1)^.	Relato de experiência^(1)^.
	Manter comunicação direta, clara e efetiva com o paciente e a família, envolvendo informações sobre o procedimento cirúrgico, processo de recuperação, sintomas e efeitos adversos esperados, planos de tratamento e tomada de decisões conjuntas sobre a terapia^(18,22,24,47,48)^.	Estudo qualitativo^(22,47,48)^, revisão narrativa da literatura^(18,24)^.
	Envolver paciente e família na educação dos cuidados que serão necessários no pós-operatório e no processo de recuperação^(9,18,22,24)^.	Revisão sistemática^(9)^, estudo qualitativo^(22)^, revisão narrativa da literatura^(18,24)^.
	Promover o acolhimento, suporte das necessidades, cuidado humanizado, encorajar o paciente ao enfrentamento do câncer e fortalecer o vínculo de confiança entre paciente, família e equipe multiprofissional^(32,47,48)^.	Estudo qualitativo^(47,48)^, revisão narrativa da literatura^(32)^.
	Considerar a necessidade de suporte psicológico e assistência social. Sempre que possível, realizar uma visita à UTI antes do procedimento como parte de orientação e diminuir a ansiedade do pós-operatório^(24,25,32)^.	Estudo de coorte^(25)^, revisão narrativa da literatura^(24,32)^.
	Promover a qualidade do sono, diminuir os barulhos e ruídos na UTI, e permitir visitas abertas dos familiares^(24,47)^.	Estudo qualitativo^(47)^, revisão narrativa da literatura^(24)^.
**Cuidados Ocupacionais à Equipe**	Manter tampa abaixada ao dar a descarga ao desprezar resíduos. Roupas contaminadas com secreções devem ser ensacadas e encaminhadas à lavanderia para lavagem separada das demais^(2,13)^.	Revisão narrativa da literatura^(2,13)^.
	Paramentação adequada durante o manuseio e eliminação dos fluídos dos drenos e urina por até 48 horas do pós-operatório. Utilizar avental impermeável, touca, luvas de procedimento, óculos de proteção e máscara cirúrgica^(1,3,5,13)^.	Relato de experiência^(1)^, estudo de caso^(3)^, revisão narrativa da literatura^(5,13)^.
	Evitar que mulheres grávidas ou que pretendam engravidar, pessoas com histórico oncológico ou imunodeprimidas prestem assistência aos pacientes submetidos à HIPEC, ou nas 48 horas de pós-operatório^(5,7)^.	Revisão narrativa da literatura^(5,7)^.

Nota: UTI – Unidade de Terapia Intensiva; NPO – nada pela via oral; HIPEC – *hyperthermic intraoperative chemotherapy*.

Com relação à categoria monitorização hemodinâmica, observa-se que a otimização do status hemodinâmico individualizado (n = 19) e a atenção ao controle rigoroso do balanço hídrico, com avaliação do volume de conteúdo em drenos, sondas e tubos (n = 14) foram os cuidados hemodinâmicos com mais citações de estudos na revisão de escopo. Ainda, preconiza-se a utilização de cateter epidural como padrão ouro para analgesia (n = 23), além da analgesia multimoldal (n = 11), para melhor controle e alívio da dor pós-operatória. Quantos aos aspectos nutricionais, observa-se a indicação de iniciar a nutrição ao paciente o mais precocemente possível (n = 14), e na mobilização, estimular exercícios e mobilização ainda no primeiro dia pós-operatório (n = 9), aos pacientes submetidos à citorredução e HIPEC durante a recuperação em UTI.

## DISCUSSÃO

A partir do mapeamento realizado dos estudos incluídos na revisão, foram identificados 72 cuidados, agrupados em 14 áreas de necessidades assistenciais para os pacientes submetidos à citorredução e HIPEC, com recuperação pós-operatória na UTI. Durante o intraoperatório, a citorredução e a utilização do

quimioterápico intra-abdominal desencadeiam alterações multiorgânicas importantes que poderão levar o paciente a apresentar instabilidades sistêmicas graves se não houver rápida intervenção assistencial da equipe^(7,26,37)^.

A ventilação mecânica prolongada influencia no tempo de permanência de internação em UTI, além dos riscos inerentes como pneumonia associada à ventilação mecânica (PAV)^(38)^. Preconiza-se a extubação assim que forem observados o despertar e melhora ventilatória^(1,28,31,33)^. A extubação precoce tem se revelado uma estratégia eficaz para a redução de tempo de internação em UTI: em estudo multicêntrico realizado no Japão, englobando 37.983 pacientes, os hospitais que apresentaram maior proporção de extubação precoce também tiveram uma menor prevalência de complicações respiratórias, assim como tiveram uma redução nos custos hospitalares^(49)^.

Após a extubação, o paciente poderá se beneficiar do uso de pressão positiva contínua nas vias aéreas (CPAP), permitindo a expansão pulmonar e consequente prevenção de atelectasias, falhas de extubação e risco de retorno à ventilação mecânica^(7,27,28)^. Em estudo realizado com pacientes com câncer de pulmão em pós-operatório, o uso de CPAP, ao ser comparado com o uso de rotina de cânula nasal, também promoveu menor dispneia e menor perda de peso um mês após a cirurgia^(50)^.

À admissão em UTI, a ressuscitação hemodinâmica deverá ser realizada com base na performance transoperatória e status das primeiras horas de recuperação: à enfermagem cabe avaliação rigorosa do padrão hemodinâmico, a partir da avaliação dos sinais vitais de pressão arterial invasiva, pressão venosa central, frequência cardíaca, perfusão periférica e rigoroso registro do balanço hídrico^(13,14,29,37,38)^. Espera-se que nas primeiras 72 horas haja uma perda de aproximadamente quatro litros de fluidos, drenados principalmente por drenos torácicos, abdominais, sondas nasogástrica e vesical, o que acarreta uma alta depleção de líquidos e consequente risco de instabilidade hemodinâmica^(2,26,27)^.

Ao manipular o conteúdo presente em drenos, deve-se manter um cuidado especial à equipe quanto ao uso de equipamentos de proteção individual nas primeiras 48 horas em UTI, a fim de evitar contato acidental direto com o quimioterápico ainda presente nos drenos e cateteres^(1,3,5,13)^. A utilização de determinados quimioterápicos no transoperatório poderá desencadear perda de função renal no paciente na UTI, exigindo-se rigorosa avaliação da função renal por meio de exames de creatinina e ureia, bem como observação do volume de diurese eliminado, sendo o ideal 1 ml/kg/h nas primeiras 72 horas de internação pós-operatória^(5,13,26,32,36)^.

O manejo da dor pós-operatória por meio da analgesia por cateter epidural foi citado pela maioria dos estudos como método mais adequado e seguro para controle e alívio da dor. O adequado controle álgico implica em recuperação pós-operatória com redução de eventos, complicações respiratórias e promoção da mobilização^(24)^. Em estudo desenvolvido com 124 pacientes na França submetidos à citorredução e HIPEC, a utilização de cateter epidural, associada à analgesia multimodal e um programa de mobilização precoce e exercícios respiratórios, demostrou redução no tempo de internação em UTI^(40)^.

Além disso, um estudo realizado em Taiwan ainda demonstrou que o controle adequado da dor é associado a uma maior qualidade de vida do paciente, assim como a um aumento na satisfação do atendimento, representando benefícios tanto para o paciente quanto para a instituição que o atende^(51)^. A maioria dos pacientes admitidos em UTI necessitará do uso de drenos específicos.

No entanto, o uso de sonda nasogástrica deverá ser avaliado individualmente quanto a sua necessidade, orientando-se a sua retirada o mais precocemente possível^(20)^. A depender da complexidade de manipulação cirúrgica e risco de íleo-paralítico, preconiza-se a permanência^(31,33)^. O jejum superior a 24 horas deverá preferencialmente ser evitado em todos os pacientes^(4,46)^. Estudo com 109 pacientes submetidos à citorredução e HIPEC em recuperação na UTI demonstrou que o jejum pós-operatório superior a 24 horas persistiu como um fator de risco para prolongação da internação em UTI e uma tendência maior ao desenvolvimento de infecções^(46)^.

A diretriz do ERAS orienta o início da nutrição preferencialmente enteral o mais cedo possível, ainda no primeiro dia de admissão em UTI, com progressão da modalidade de dieta conforme aceitação e condição clínica e cirúrgica do paciente^(8,19,20,28)^. A nutrição enteral precoce reduz o risco de complicações como translocação bacteriana e desnutrição do paciente crítico^(7,38)^. Todavia, na presença de complicações pós-operatórias como íleo-paralítico, ressecção de órgãos intra-abdominais, fístulas e sepse, deve-se reavaliar a administração de dieta enteral^(8)^. Em face da impossibilidade de início da nutrição enteral, seja por via oral ou por sonda, a equipe multiprofissional deve reavaliar a administração de nutrição parenteral total, principalmente naqueles pacientes sem aporte dietético até três dias^(7,8,37,38,44)^.

Infeções desenvolvidas no período de recuperação em UTI estão diretamente relacionadas a morbidade, prolongação de internação e custos hospitalares^(23)^. Pesquisa chinesa com 482 pacientes submetidos à citorredução e HIPEC demonstrou que os sítios mais comuns de infecção observados foram acessos venosos centrais (8,1%) e infecção abdominopélvica relacionada ao sítio cirúrgico (5,2%), além de ser fator de risco independente para perda de sangue no perioperatório e ascite^(52)^. Dessa forma, torna-se imprescindível que as medidas de barreiras às infecções relacionadas à assistência à saúde sejam preconizadas como rotina em qualquer UTI, como adequada higiene das mãos dos profissionais e educação ao familiar e paciente, adequada manipulação de acessos centrais, medidas de prevenção de pneumonia associada à ventilação mecânica (PAV) e manutenção adequada do sistema fechado de drenagem vesical^(18,21,33)^.

A enfermagem deve manter rigoroso controle glicêmico, almejando o alvo de glicemia entre 140 a 180 mg/dL^(4,8,19,20,29)^. É frequente o desenvolvimento de hiperglicemias devido ao intenso processo inflamatório nos pacientes submetidos à HIPEC. Assim, deve-se atentar para a necessidade de suplementação insulínica^(9,19,26)^. Logo à admissão, é comum a hipotermia, principalmente nos pacientes submetidos à modalidade de cirurgia aberta. Portanto, é necessário um rigoroso controle de temperatura, com meta acima de 36°C, e avaliação da necessidade de instalação de sistemas de aquecimento como mantas térmicas ou fluidos aquecidos^(4,10,14,30,32)^.

Devido à condição oncológica, simultaneamente à HIPEC, os pacientes poderão apresentar anormalidades no perfil de coagulação, com predisposição à formação de trombos, sob o risco de desenvolvimento de trombose venosa profunda^(26)^. Medidas de profilaxia farmacológica, como a utilização de heparina não fracionada pela equipe médica na ausência de contraindicação e sangramentos, em associação ao uso de meias compressoras e botas de retorno venoso, auxiliam na diminuição do risco de eventos trombóticos^(2,5,7,8)^. Além do cuidado físico, especial atenção deve ser dada à saúde mental tanto de paciente como família. Preconiza-se envolver ambos, em conjunto com a equipe multiprofissional, na tomada de decisões terapêuticas, bem como oferecer informações completas e contínuas sobre cuidados e possíveis riscos inerentes à cirurgia^(22,47,48)^. A adequada comunicação entre equipe, paciente e família reduz, principalmente para o paciente, sintomas como ansiedade e estresse durante o processo de recuperação^(24)^.

De todos os cuidados aqui discutidos, a sua maioria está presente na atual diretriz do protocolo ERAS^(8)^. Frente a desfechos positivos de recuperação pós-operatória, a utilização das intervenções discutidas no protocolo ERAS proporciona redução da média dos custos hospitalares devido à diminuição do tempo de internação hospitalar (7 ± 1.1 dias), quando comparada à sua não utilização (10 ± 4.5 dias), como demonstrado em estudo desenvolvido na Turquia^(19)^.

Apontam-se, no presente estudo, algumas limitações, como a de que a maioria dos estudos são revisões narrativas da literatura, apresentando consequentemente baixo nível de evidência. Durante a realização da revisão de escopo, os autores observaram poucos estudos com desenho metodológico mais robustos com informações precisas sobre cuidados pós-operatórios em UTI, o que repercutiu na inclusão de revisões a fim de verificar por completo o que havia na literatura sobre os cuidados assistenciais a este perfil de pacientes. Ainda, poucos eram os estudos com cuidados direcionados à assistência de enfermagem, sendo mais presentes em artigos a temática de cuidados generalizada, medicina ou revisões.

Evidencia-se a necessidade de publicação de estudos referentes aos cuidados de enfermagem aos pacientes em pós-operatório de citorredução e HIPEC com desenho metodológico de melhor qualidade e voltados à enfermagem. Todavia, durante a avaliação dos estudos a serem incluídos, observou-se um único estudo brasileiro sob o formato de revisão narrativa com abordagem de cuidados pós-operatórios em UTI após a citorredução e HIPEC. Ainda, outro estudo brasileiro abordava unicamente cuidados referentes ao jejum e nutrição pós-operatória.

Assim, o presente artigo é a primeira revisão de escopo sob a ótica da enfermagem, que mapeou as principais práticas assistenciais de cuidados a serem realizados na UTI para pacientes submetidos à citorredução e HIPEC. A equipe de enfermagem deve saber quais as necessidades que esses pacientes têm no pós-operatório e repensar suas práticas, aplicando as evidências científicas na rotina do cuidado, a fim de proporcionar uma assistência pautada na literatura, em prol da adequada recuperação e segurança do paciente e equipe.

## CONCLUSÃO

A partir da realização da revisão de escopo, foi possível mapear 72 cuidados presentes na literatura científica para pacientes submetidos à citorredução e HIPEC. Esses cuidados foram agrupados em 14 áreas assistenciais, para facilitar a compreensão das atividades desempenhadas no pós-operatório. Observou-se que a assistência de enfermagem é semelhante àquela para qualquer paciente submetido a cirurgia de grande porte que se recupera na UTI, com algumas particularidades para a equipe que presta os cuidados nas primeiras 48h. Dos 42 estudos selecionados, apenas dois eram artigos brasileiros abordando a temática, sendo um de revisão narrativa, com cuidados pós-operatórios em UTI, e o outro um estudo de caso.

Dentre os cuidados mapeados, verificou-se uma concentração maior de cuidados referentes às temáticas de monitorização hemodinâmica e ventilatória, atenção à dor, cuidados nutricionais e de mobilização. Evidenciou-se a necessidade de estudos com metodologia mais robusta quanto à evidência científica da segurança desses cuidados, principalmente direcionados à equipe de enfermagem. Conhecer as práticas assistenciais a serem implementadas em UTI a esse perfil de pacientes possibilita um cuidado seguro, cientificamente embasado, que promova a adequada recuperação.
